# Supplementary material for: Exploring the Diversity of Biofilm Formation by the Food Spoiler Brochothrix thermosphacta
Source: Microorganisms. 2022 Dec 15;10(12):2474. doi: 10.3390/microorganisms10122474 (PMC9785830; doi:10.3390/microorganisms10122474)
Supplement: Supplementary file 1 [file microorganisms-10-02474-s001.zip › microorganisms-2089188-supplementary.pdf]

Exploring the diversity of biofilm formation by the food spoiler *Brochothrix thermosphacta* (supplementary data)

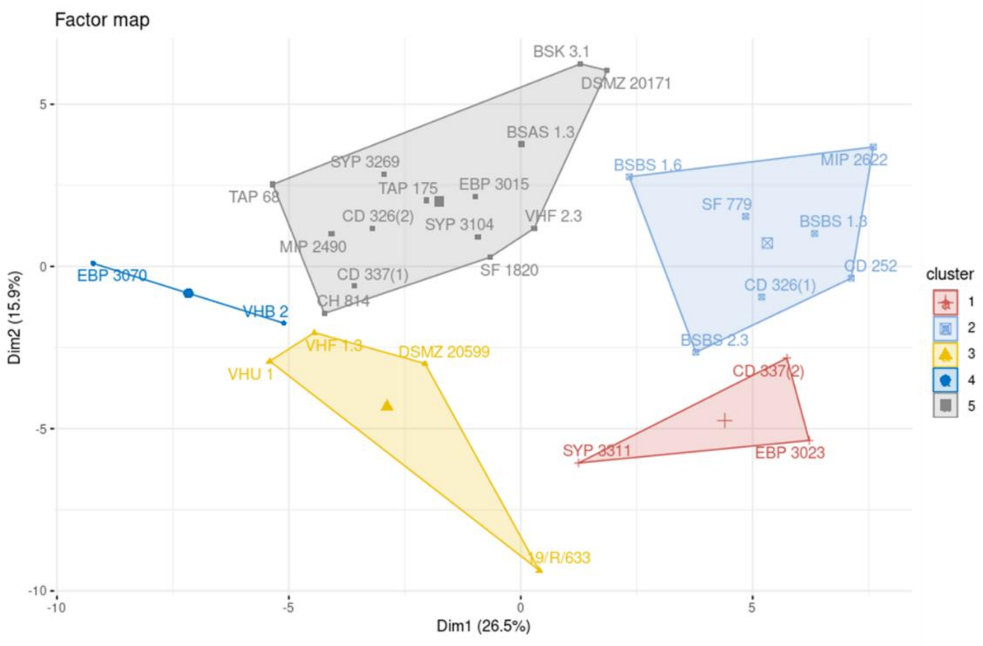

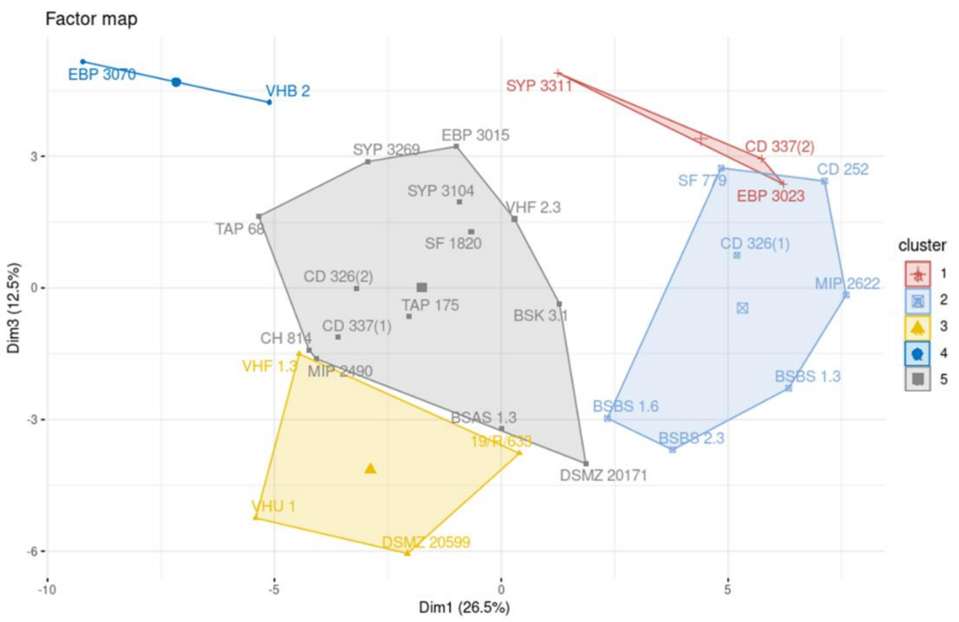

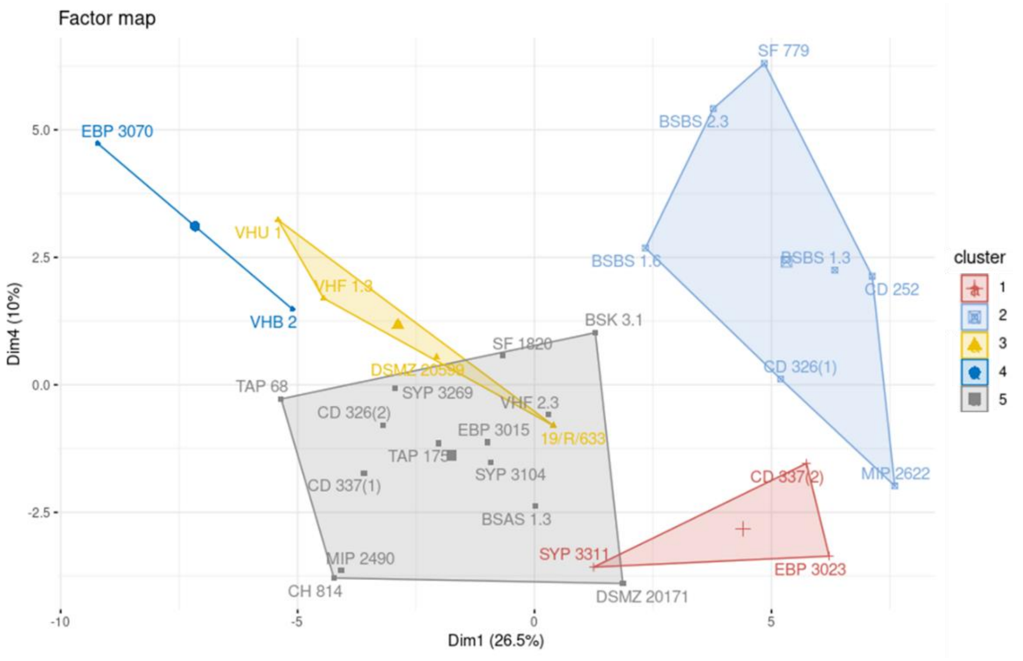

Supplementary figure 1. Representation of the HCPC clusterisation tree based on ACP. Those 5 clusters were projected on the first 4 dimensions of the ACP representing 65% of the global variation to identified which parameters could characterized those clusters. The dimension 1, 26.5% of the global variance, separated cluster 4 from the clusters 3, 5 and from 1, 2. This dimension was mostly influenced by parameters extracted at 24h, such as number of cells, biofilm surface area, biofilm thickness and biofilm volume (S2), suggesting a different or similar architecture linked with cell concentration and volume of the mature biofilm between those clusters. The dimension 2, 15.9% of the global variance, separated the cluster 4 from the clusters 1, 3 and from the clusters 2, 5. Dimension 2 was influenced by parameters extracted at 24 h, such as biofilm local density, biofilm outer surface and biofilm surface local roughness (S3).The dimension 3, 12.5% of the global variance, separated the cluster 3 from the clusters 1, 4 and from the clusters 2, 5. This dimension was mostly influenced by parameters extracted at 2h such as surface per subtract area, shape volume, biofilm height and biofilm local density (S4), suggesting a different or similar architecture of the early biofilm between those clusters. The dimension 4, 10% of the global variance, separated the clusters 1, 5 from the clusters 2 ,3 and 4. Dimension 4 was influenced by parameters extracted at 2 h and 24 h (S5), such as thickness of the biofilm at 2 h, shape roughness on the images at 2 h, outer surface per substrate at 24 h and the local substrate area at 24 h.

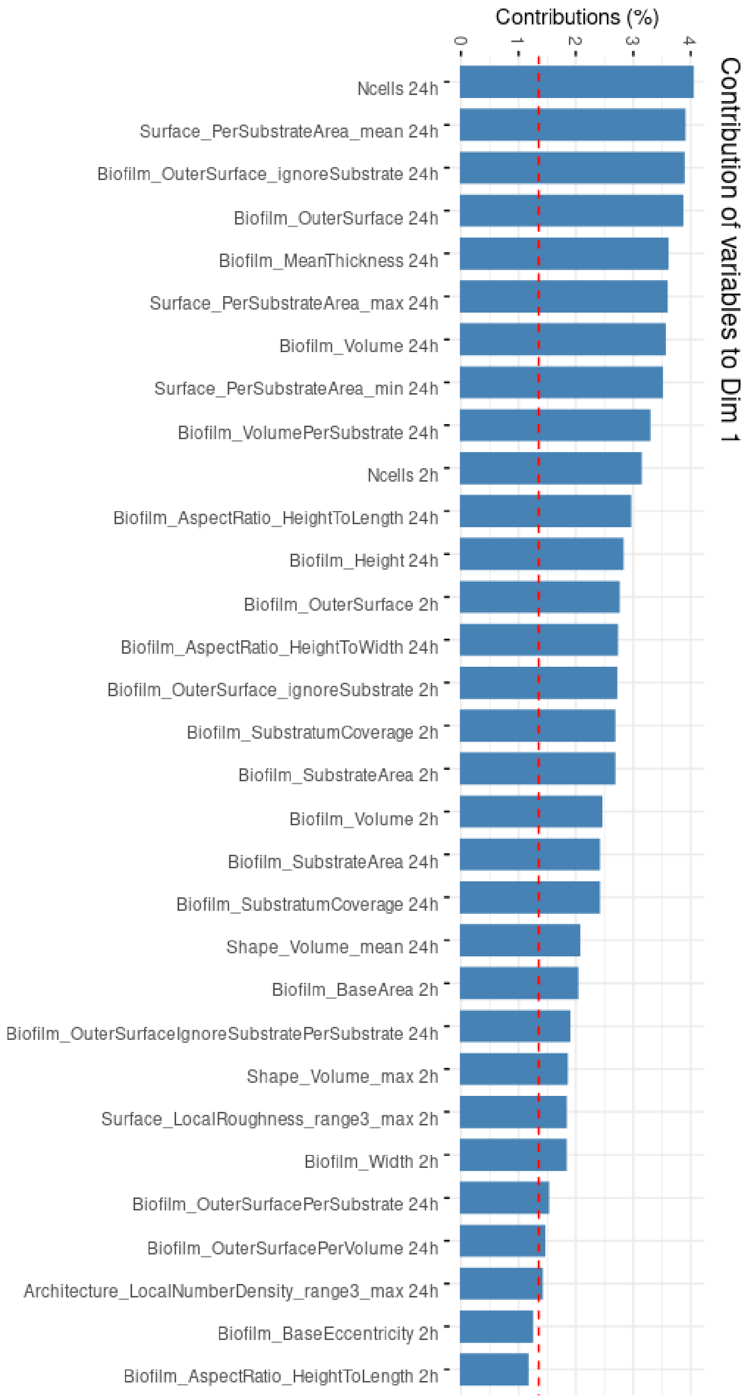

Supplementary figure 2. Parameters contribution of variables to dimension 1. To achieve the extraction from the images, the default setting was used excepted for 2.3 Denoising where "suppress floating cells by median filtering along z" was activated. For 2.4 Thresholding, the "thresholding method Otsu", with 3 classes assign class 2 to background (2 h incubated biofilm), and "Otsu 2 classes" (24 h incubated biofilm) was used, both with 0.5 sensitivity. For 2.5 Objects declumping: a cube side length of 10 was used. For the parameter calculation, "Surface properties, option: range [vox]", "Substrat area", "Global biofilm properties" and "Local density, option: range [vox]" were selected.

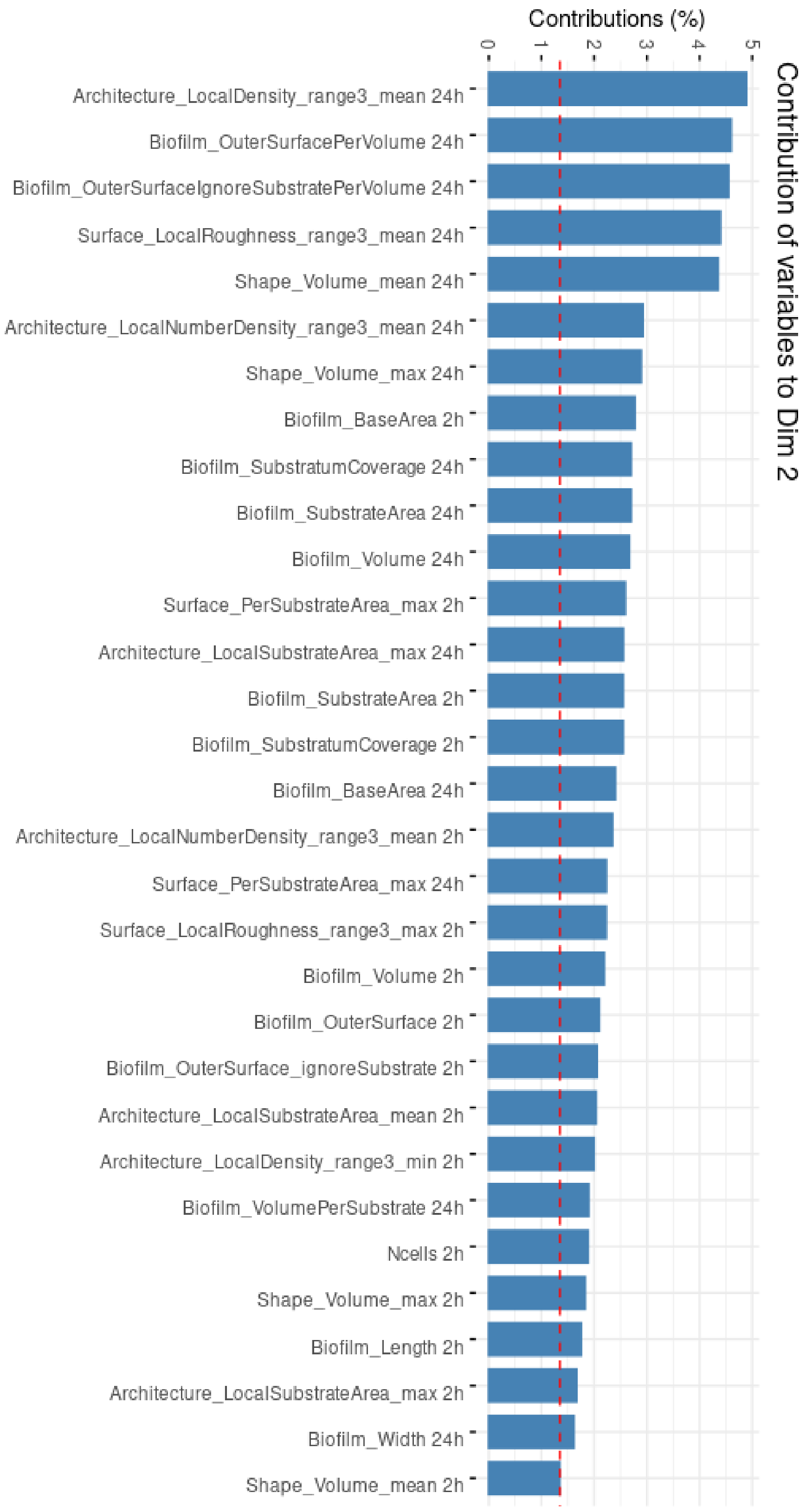

Supplementary figure 3. Parameters contribution of variables to dimension 2. To achieve the extraction from the images, the default setting was used excepted for 2.3 Denoising where "suppress floating cells by median filtering along z" was activated. For 2.4 Thresholding, the "thresholding method Otsu", with 3 classes

assign class 2 to background (2 h incubated biofilm), and “Otsu 2 classes” (24 h incubated biofilm) was used, both with 0.5 sensitivity. For 2.5 Objects declumping: a cube side length of 10 was used. For the parameter calculation, “Surface properties, option: range [vox]”, “Substrat area”, “Global biofilm properties” and “Local density, option: range [vox]” were selected.

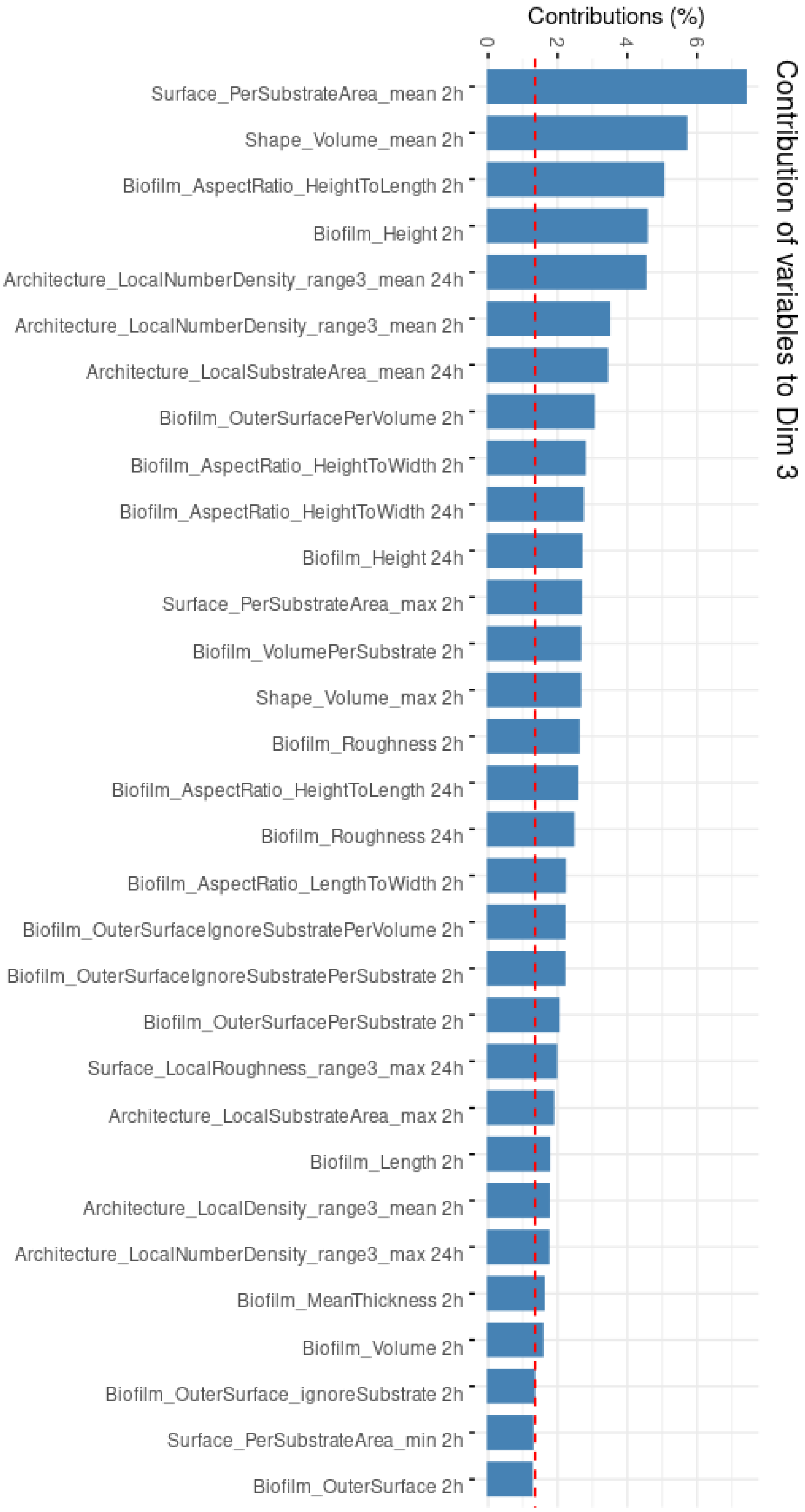

Supplementary figure 4. Parameters contribution of variables to dimension 3. To achieve the extraction from the images, the default setting was used excepted for 2.3 Denoising where "suppress floating cells by median filtering along z" was activated. For 2.4 Thresholding, the "thresholding method Otsu", with 3 classes assign class 2 to background (2 h incubated biofilm), and "Otsu 2 classes" (24 h incubated biofilm) was used, both with 0.5 sensitivity. For 2.5 Objects declumping: a cube side length of 10 was used. For the parameter calculation, "Surface properties, option: range [vox]", "Substrat area", "Global biofilm properties" and "Local density, option: range [vox]" were selected.

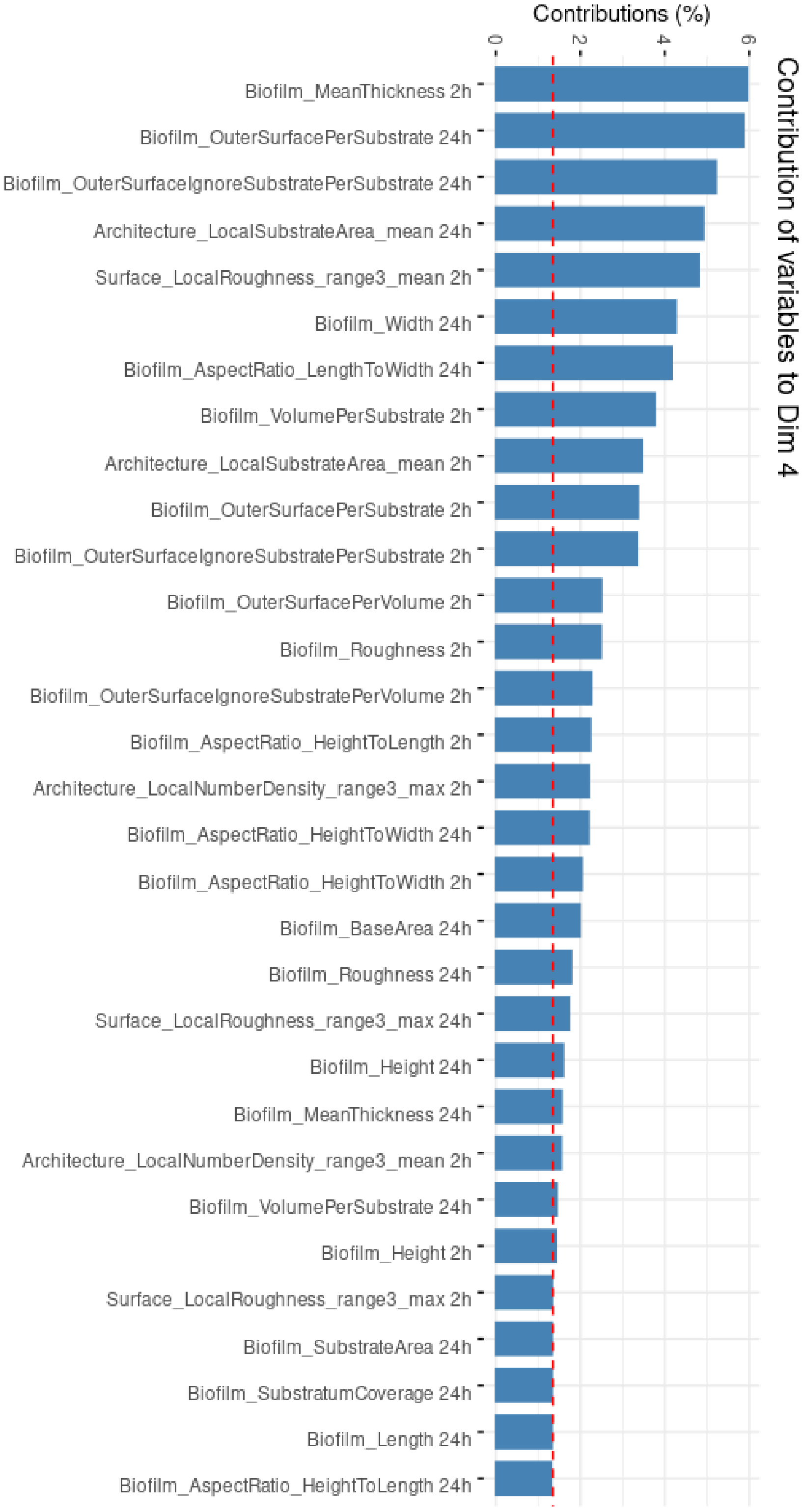

Supplementary figure 5. Parameters contribution of variables to dimension 4. To achieve the extraction from the images, the default setting was used excepted for 2.3 Denoising where "suppress floating cells by median filtering along z" was activated. For 2.4 Thresholding, the "thresholding method Otsu", with 3 classes assign class 2 to background (2h incubated biofilm), and "Otsu 2 classes" (24 h incubated biofilm) was used, both with 0.5 sensitivity. For 2.5 Objects declumping: a cube side length of 10 was used. For the parameter calculation, "Surface properties, option: range [vox]", "Substrat area", "Global biofilm properties" and "Local density, option: range [vox]" were selected.

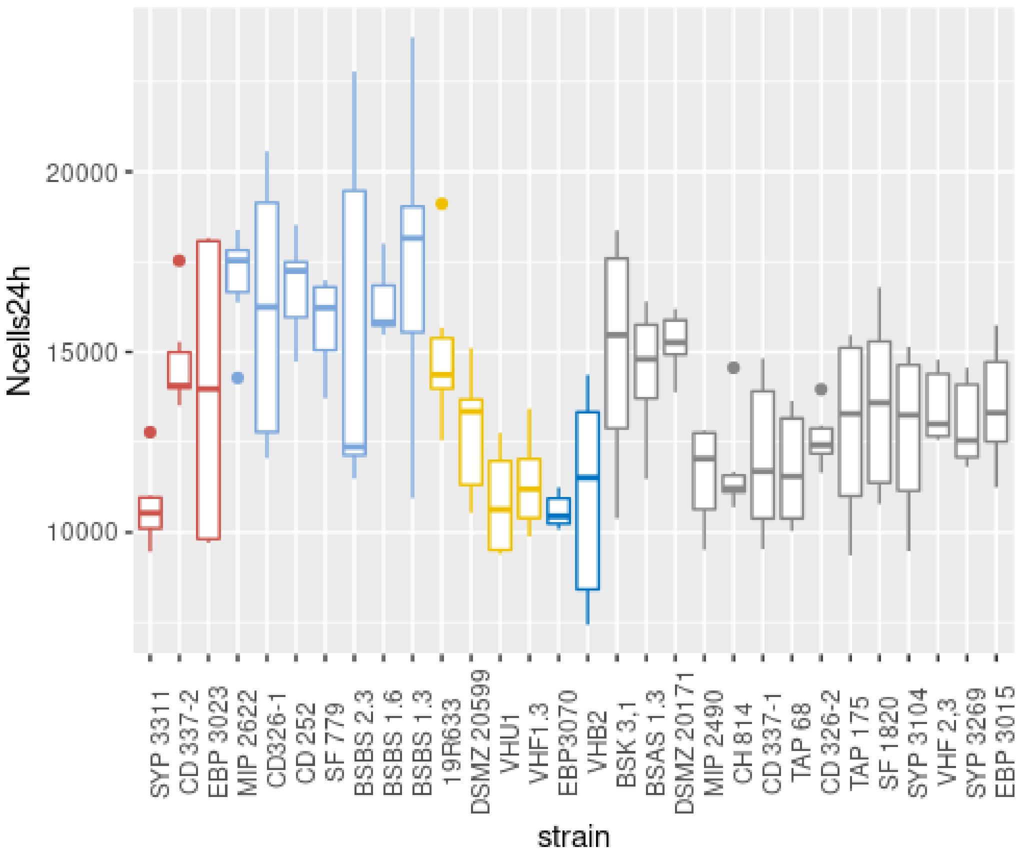
Ncells24h
20000
15000
10000
SYP 3311
CD 337-2
EBP 3023
MIP 2622
CD326-1
CD 252
SF 779
BSBS 2.3
BSBS 1.6
BSBS 1.3
19R633
DSMZ 20599
VHU1
VHF1.3
EBP3070
VHB2
BSK 3,1
BSAS 1.3
DSMZ 20171
MIP 2490
CH 814
CD 337-1
TAP 68
CD 326-2
TAP 175
SF 1820
SYP 3104
VHF 2,3
SYP 3269
EBP 3015
strain

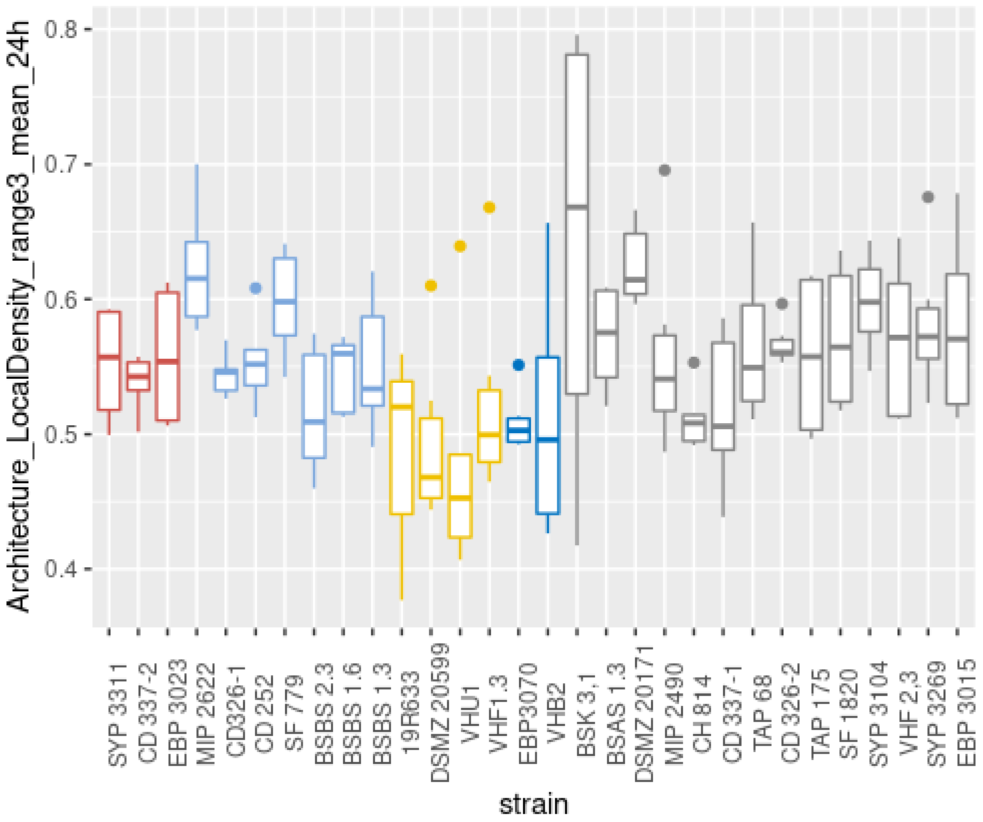
Architecture_LocalDensity_range3_mean_24h
0.8
0.7
0.6
0.5
0.4
SYP 3311
CD 337-2
EBP 3023
MIP 2622
CD326-1
CD 252
SF 779
BSBS 2.3
BSBS 1.6
BSBS 1.3
19R633
DSMZ 20599
VHU1
VHF1.3
EBP3070
VHB2
BSK 3,1
BSAS 1.3
DSMZ 20171
MIP 2490
CH 814
CD 337-1
TAP 68
CD 326-2
TAP 175
SF 1820
SYP 3104
VHF 2,3
SYP 3269
EBP 3015
strain

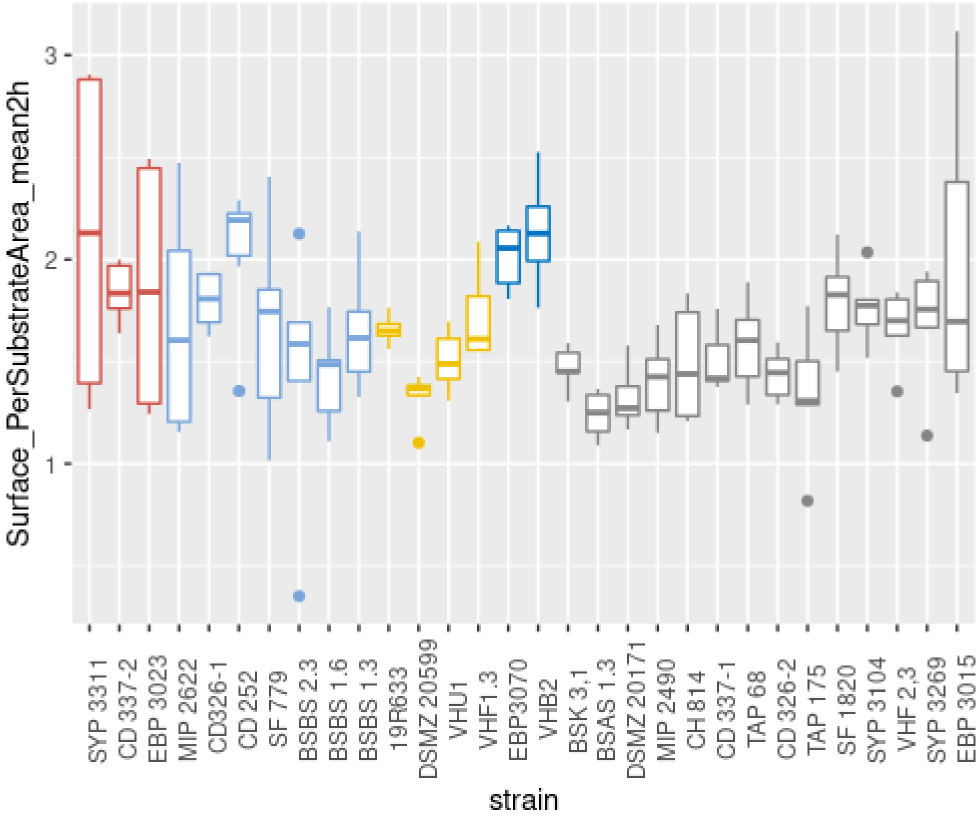

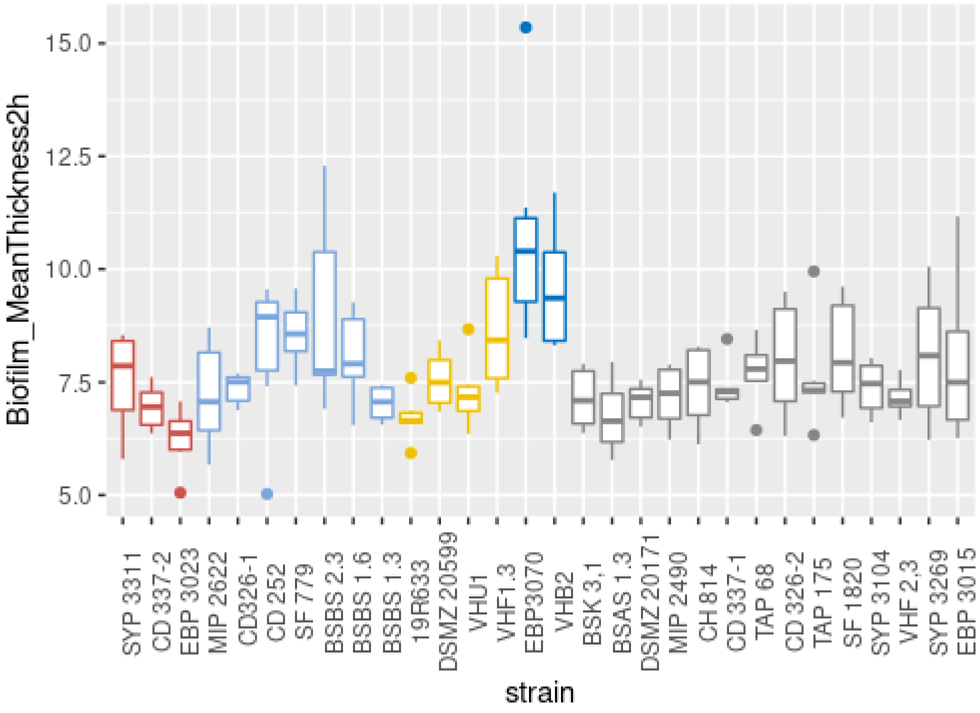

Supplementary Figure 6. ANOVA test on biofilm number of cells and biofilm density at 24h and on biofilm surface area per substrate area and biofilm thickness at 2h. Each cluster is labeled by specific color (see HCPC clusterisation figure 3) and the p value are respectively $p=1.35x10^{-7}$, $p=4.90x10^{-4}$, $p=1.21x10^{-5}$, $p=9.80x10^{-5}$. Those 5 clusters are compared using the top structural biofilm parameters of the 4

dimensions extracted by BiofilmQ, for 2 h and 24 h with an ANOVA test. The cluster 1 shown a low biofilm thickness and a high biofilm surface per substrate area at 2h, at 24h this cluster shown a high cell concentration and a biofilm density equivalent to the cluster 2 and 5. The cluster 2 shown a medium biofilm thickness and biofilm surface per substrate area at 2h, at 24h this cluster shown the higher cell concentration and a biofilm density equivalent to the cluster 5 and 1. The cluster 3 shown a low thickness and biofilm surface per substrate area at 2h and a low number of cells, biofilm density at 24h.The cluster 4 biofilm shown the higher biofilm thickness and biofilm surface per substrate area at 2h while at 24h this cluster shown a lower cell concentration and the lower biofilm density. The cluster 5 had a low thickness and a low biofilm surface per substrate area at 2h, at 24h a medium cell concentration and a biofilm density equivalent to the cluster 1 and 2.
